# Intervention effects of different brands of botulinum toxin type A on Meige syndrome with anxiety symptoms

**DOI:** 10.3389/fneur.2026.1692145

**Published:** 2026-05-07

**Authors:** Yunyu Tang, Ruen Liu

**Affiliations:** Department of Neurosurgery, Peking University People’s Hospital, Beijing, China

**Keywords:** anxiety, botulinum toxin type A, Meige syndrome, sleep quality, treatment

## Abstract

**Background:**

Patients with Meige syndrome are often affected by anxiety symptoms, and drug therapy is the primary treatment option, with botulinum toxin A being the most frequently used medication. This study analyzes the intervention effects of different brands of botulinum toxin A on Meige syndrome with anxiety symptoms. The findings aim to provide a theoretical basis for the rational selection of clinical treatment options.

**Methods:**

A retrospective analysis was conducted on 148 patients treated between January 2021 and December 2023. Patients were divided into two groups based on the BoNT-A brand received: Chinese botulinum toxin type A (Hengli) group (*n* = 80) and onabotulinumtoxinA (Botox) group (*n* = 68). Both groups received local injections at the sites of muscle spasms. The outcomes included the degree of spasm relief, duration of efficacy, scores on the Self-Rating Anxiety Scale (SAS) and Self-Rating Depression Scale (SDS), polysomnography parameters [sleep efficiency (SE), total sleep time (TST), sleep latency (SL), number of wakefulness (AI), rapid eye movement sleep time (REMS)], and adverse reactions.

**Results:**

There were no statistically significant differences in the total remission rate and efficacy maintenance time between the two groups (*p* > 0.05). The differences in the scores of the SAS, SDS, SE, TST, SL, AI, and REMS before and after treatment between the two groups were not statistically significant (*p* > 0.05). There was no statistically significant difference between the two groups when comparing the incidence of adverse reactions such as incomplete eyelid closure, facial muscle weakness, and drooping of the corners of the mouth during expression muscle activity (*p* > 0.05).

**Conclusion:**

Local injection of botulinum toxin type A is a safe and effective treatment for Meige syndrome with anxiety symptoms, and there is no significant difference in the efficacy, duration of action, and improvements in psychological state and sleep quality between different brands of botulinum toxin type A. Therefore, clinical practice should be based on practical considerations to reasonably select the appropriate brand of botulinum toxin type A for treatment.

## 1 Introduction

Meige syndrome is a craniofacial dystonia with blepharospasm and orofacial dystonia as the main clinical manifestations, which can also be accompanied by dystonia in other parts of the neck, trunk, and limbs, and therefore has also been named segmental dystonia (1). Meige syndrome is categorized into four types according to the site of involvement: blepharospasm type, orofacial dystonia type, blepharospasm combined with orofacial dystonia type, and other types (combining dystonia in other parts of the body based on the first three types), among which blepharospasm combined with orofacial dystonia type is also known as the complete type of Meige syndrome (2). According to a survey, Meige syndrome, as a relatively rare neurological disorder, has an incidence rate of 59/1 million (3). The main clinical manifestations of patients are involuntary contraction of local muscles in the face, and in severe cases, visual impairment and swallowing disorders may occur, which seriously affect normal life functions, appearance, and social activities, thus greatly affecting patients’ self-esteem and self-confidence and leading to anxiety and other negative emotions (4). The study of Ouyang J et al. (5) found that 40–60% of patients with Meige syndrome had anxiety symptoms and that anxiety symptoms could interact with disease symptoms, leading to the aggravation of the patients’ conditions. Therefore, it is important to adopt effective and scientific treatment programs in time to alleviate patients’ conditions and improve their quality of life.

Traditionally, the treatment of Meige syndrome mainly consists of oral treatment with antiepileptic drugs, sedative drugs, traditional Chinese medicine, and acupuncture, but the therapeutic effect is not obvious in most patients (6). At the beginning of the disease treatment, the condition was slightly relieved, but the efficacy of the treatment gradually waned, and most of the patients could not tolerate the adverse effects of the relevant antiepileptic drugs and sedative drugs (7). In addition to the above treatments, local injection of botulinum toxin type A, a highly toxic neuroexotoxin produced by the bacterium *Clostridium botulinum*, has been used in the treatment of Meige syndrome (8), mainly by inhibiting the release of acetylcholine from the presynaptic membrane of the motor nerve endings, which leads to muscle flaccid paralysis. Local botulinum toxin injection triggers germination of motor nerve endings, altered motor functional connectivity, and abnormal neuromuscular conduction pathways are weakened, thereby relieving patients’ clinical symptoms (9). Botulinum toxin type A has been widely used in clinical practice for an expanding range of indications in neurological disorders such as restrictive dystonia, facial dystonia, post-stroke sequelae, and migraines, and has been progressively generalized to Meige syndrome with anxiety symptoms (10). A study by Yazdanpanah G et al. (11) found that botulinum toxin type A was effective in treating myasthenia gravis while reducing the degree of wrinkles around the eyes and restoring normal visual and swallowing functions, allowing patients to return to normal life. However, there are different brands of botulinum toxin A available, and there is heterogeneity in the molecular composition, diffusion characteristics, and immunogenicity of different brands of botulinum toxin A. Whether the characteristics of a particular brand will have an impact on the efficacy of patients with Meige syndrome with anxiety symptoms is still not clinically conclusive.

Furthermore, sleep disturbances are frequently reported in patients with Meige syndrome, potentially due to the involvement of limbic and dopaminergic pathways that regulate both motor control and sleep architecture. Chronic facial spasms and associated pain may also contribute to sleep fragmentation. Therefore, evaluating sleep parameters through polysomnography provides a comprehensive assessment of treatment impact on both motor and non-motor symptoms.

Based on this, this article analyzes the effects of two different brands of botulinum toxin type A, Hengel and Botox, on Meige syndrome with anxiety symptoms in a retrospective cohort study, which is expected to guide the development of an optimal treatment plan to enhance the clinical outcomes of the patients, to improve the symptoms of facial spasms and anxiety, and to improve their quality of life and social functioning, as well as to promote the advancement of the field in the treatment of Meige syndrome.

## 2 Materials and methods

### 2.1 Ethics statement

This study was approved by our Institutional Review Board and Ethics Committee. Given that this study was retrospective and only de-identified patient data were used, informed consent was not required as there was no risk or adverse effect on patient care. This waiver is in line with regulatory and ethical guidelines related to retrospective studies.

### 2.2 Study design

This retrospective study included 148 patients with Meige syndrome with anxiety symptoms who were treated at our hospital between January 2021 and December 2023. The patients were divided into two groups according to their medication regimen: the onabotulinumtoxinA (Botox) group (*n* = 68) and the Chinese botulinum toxin type A (Hengli) group (*n* = 80).

### 2.3 Inclusion criteria

Inclusion criteria included: (1) all meeting the diagnostic criteria of Meige syndrome (12) and having a score of ≥50 on the Self-Assessment Scale for Anxiety (SAS) (13); (2) age ≥18 years old, regardless of gender; (3) good communication and communication skills; (4) no previous history of anxiety, depression, and sleep disorders; (5) no recent major life events; and (6) complete clinical data.

Exclusion criteria included: (1) patients with previous secondary Meige syndrome due to cephalo-facial trauma, vascular factors, tumors, inflammation, medications, congenital malformations, and other neurological disorders; (2) patients who have been diagnosed with facial muscle spasm, myasthenia gravis, trigeminal neuralgia, and Lambert–Eaton syndrome; (3) patients who are allergic to the drugs used in the study; (4) pregnant or lactating patients; (5) patients with skin infection at the injection site; and (6) patients with acute fever or combined severe cardiac, pulmonary, hepatic, renal, and hematopoietic system diseases.

### 2.4 Treatment methods

The Hengli group was given injectable botulinum toxin type A (trade name: Hengli, Lanzhou Biological Products Research Institute Co., Ltd., National Drug License: S10970037) for local injection, and the Botox group was given injectable botulinum toxin type A (trade name: Botox, Allergan Pharmaceuticals Ireland, Imported Drugs Registration Certificate no: S20120067/S20172005) for local injection. Both drugs were stored frozen at −5 °C and diluted to 2.5 U/0.1 mL by adding 0.9% sodium chloride injection and were injected locally at the site of muscle spasms, with a dose of 0.1 ~ 0.2 mL per spot. The site of injection was determined according to the distribution of the muscle groups involved in the spasms, including the inner part of the upper and lower eyelids, the arch of the eyebrow, the outer canthus, the zygomaticus major muscle, the zygomaticus minor muscle, the interbrow orbicularis oris, oris, the biting muscle, the smiling muscle, the lifting upper lip, and the nasal wing muscle. The injection site was determined according to the distribution of the muscles involved in the spasms. In the case of lateralis muscle spasm with tinnitus, the stapedius muscle injection was added, and in the case of Meige syndrome with blepharospasm, the levator palpebrae superioris muscle injection was added. For severe cases of lateral facial muscle spasm, the injection site was selected bilaterally, and half of the dose was injected into the expression muscle on the healthy side. The total dose per treatment session ranged from 25 to 50 U per side, depending on the number and severity of muscle groups involved. The most frequently injected muscles were the orbicularis oculi, corrugator supercilii, procerus, zygomaticus major, orbicularis oris, and mentalis muscles, consistent with standard injection patterns for craniofacial dystonia. Scrubbing and massaging of the injection site were prohibited for 24 h. Return visits were performed at 1 week, 4 weeks, 3 months, 6 months, and 12 months after injection.

### 2.5 General data collection

General demographic data of the patients were collected through the medical record system, including age, gender, body mass index, education grade, number of muscle sites involved at first onset, hypertension [consistent with the “The Japanese Society of Hypertension Guidelines for Self-monitoring of Blood Pressure at Home (Second Edition)” (14) diagnostic criteria for moderate to high blood pressure], diabetes mellitus (eligible) “Application of the Chinese Expert Consensus on Diabetes Classification in clinical practice” (15) diagnostic criteria for diabetes mellitus in, Hyperlipidemia [meets “Report of the Japan Atherosclerosis Society (JAS) Guideline for Diagnosis and Treatment of Hyperlipidemia in Japanese adults” (16) diagnostic criteria for moderate to high blood cholesterol], course of the disease and grading of spasm intensity [grade 0: no spasticity; grade 1: mild spasm can be provoked by external stimuli; grade 2: tremors can occur in the absence of stimuli; grade 3: pronounced spasm and the presence of mild functional impairment; and grade 4: severe spasm and functional impairment (17)].

These metabolic and cardiovascular variables were collected to assess baseline comparability between groups, as they may influence treatment response or be associated with comorbid conditions that affect quality of life and psychological state, although they are not directly implicated in the primary pathophysiology of Meige syndrome.

### 2.6 Assessment of the degree of spasticity relief

Three months after injection, the degree of spasm relief was assessed in both groups, complete relief: spasm intensity decreased from grade 3 or 4 to grade 0; obvious relief: spasm intensity decreased from grade 3 or 4 to grade 1 or 2; partial relief: spasm intensity decreased from grade 4 to grade 3 or grade 2 to grade 1; and no relief: spasm intensity did not improve (18). Total remission rate = (complete remission + significant remission + partial remission) number of cases/total number of cases × 100%.

### 2.7 Evaluation of efficacy maintenance time

The two groups of patients were reviewed at 3 months after the injection. Spasm symptom relief indicates the existence of the efficacy of the treatment, and the maintenance time for the efficacy of the two groups was counted.

### 2.8 Psychological state assessment

Before and 3 months after the injection, the depression and anxiety grades of the two groups of patients were assessed using the Self-assessment Depression Scale (SDS) and SAS. The SDS scale also contains 20 items, and each item adopts a 4-grade scoring method, and the scores of the 20 items were added to obtain the total crude score, and then the total crude score was multiplied by 1.25 and rounded up to the nearest whole number to obtain the standard score, and the cutoff value of the standard score is 53, 53 to 62 is mild depression, 63 to 72 is severe depression, and 72 or more is severe depression. The SAS scale also contains 20 items; each item adopts a 4-grade scoring method, summing the scores of the 20 items to get the total crude score, and then multiplying the total crude score by 1.25 and taking the integer part to get the standard score, with a cutoff value of the standard score of 50 points, 50 to 59 points for mild anxiety, and 50 to 59 points for severe anxiety. Anxiety levels ranging from 50 to 59 are classified as mild, 60 to 69 as moderate, and 70 or more as severe (19).

### 2.9 Assessment of polysomnographic monitoring parameters

Polysomnography was performed on the patients before and 3 months after the injection using Ampoland NT000 polysomnography (Amplaid, USA); sleep efficiency (SE) is the ratio of the total sleep time (TST) to the time in bed during the detection process. TST is the true sleep duration during the detection process. Sleep latency (SL) is the length of time from the time the patient went to bed to the time when the first sleep segment appeared. Awakenings (AI) are the number of awakenings ≥15S per hour during the sleep process. Rapid eye movement sleep time (REMS) is the number of awakenings ≥15S per hour during the sleep process. Wakenings (REMS) the number of awakenings ≥15S per hour during the sleep process. AI is the number of awakenings ≥15S per hour during sleep, while REMS is the length of REM sleep during testing.

### 2.10 Assessment of adverse reactions

Adverse reactions, such as incomplete eyelid closure, facial muscle weakness, and drooping of the corners of the mouth during expression muscle activity, were counted during treatment for both groups. The incidence rate of adverse reactions = the number of cases in which adverse reactions occurred/the total number of cases×100%.

### 2.11 Statistical methods

SPSS25.0 statistical software was used to analyze the data, and the count data were expressed as n or [n (%)], and the χ2 test was adopted; the measurement data conforming to normal distribution were expressed as (x̅ ± s), and the t-test was adopted. Differences were considered statistically significant at a *p*-value of < 0.05.

## 3 Results

### 3.1 Comparison of basic information of patients in the two groups

Comparison of the basic information of the two groups of patients in terms of age, gender, body mass index, education grade, site of first onset, hypertension, diabetes, hyperlipidemia, site of first onset, duration of the disease, and spasm intensity grading, the difference is not statistically significant (*p* > 0.05), suggesting that the groups are comparable, see Table 1.

**Table 1:** Comparison of basic information of patients in two groups (Hengli group vs. Botox group).

Index		Hengli group (*n* = 80)	Botox group (*n* = 68)	*χ*^2^/*t* value	*p-*value
Age (years)		64.72 ± 10.84	65.16 ± 10.92		
Sex (*n*)	Male	48	39	0.106	0.744
	Female	32	29		
Body Mass index (kg/m^2^)		21.78 ± 3.20	21.85 ± 3.34	0.130	0.897
Educational attainment (*n*)	Middle school or below	51	45	0.095	0.758
	High school or above	29	23		
High blood pressure (*n*)		8	5	0.321	0.571
Diabetes (*n*)		6	3	0.614	0.433
Hyperlipidemia (*n*)		7	6	0.000	0.987
Number of muscle sites involved at first onset		2.10 ± 0.45	2.24 ± 0.56	1.686	0.094
Duration of illness (months)		13.20 ± 3.24	13.46 ± 3.30	0.482	0.630
Spasm Intensity Grading (*n*)	Grade 1	19	13	0.491	0.921
	Grade 2	24	21		
	Grade 3	20	18		
	Grade 4	17	16		

### 3.2 Comparison of the degree of spasm relief between the two groups of patients

The total remission rate is an important index for evaluating clinical efficacy, and the higher the total remission rate, the better the efficacy. The total relief rate of the two groups (93.75% vs. 95.59%) is not statistically significant (*p* > 0.05), which indicates that two different brands of botulinum toxin A can effectively reduce the spasm symptoms of patients with Meige syndrome with anxiety symptoms, see Table 2.

**Table 2:** Comparison of the degree of spasm relief between the two groups.

Index	Hengli group (*n* = 80)	Botox group (*n* = 68)	*χ*^2^ value	*p-*value
Full remission (*n*)	18	13		
Markedly reduce (*n*)	38	40		
Partial mitigation (*n*)	19	12		
Unmitigated (*n*)	5	3		
Overall mitigation rate [*n* (%)]	75 (93.75)	65 (95.59)	0.243	0.622

### 3.3 Comparison of efficacy maintenance time between the two groups of patients

Efficacy maintenance time is an important indicator to assess the pharmacokinetic and pharmacodynamic properties of drugs *in vivo*, and the longer the efficacy maintenance time, the slower the metabolism and elimination of the drug, and the longer-lasting the efficacy of the drug. When comparing the efficacy maintenance time of 3 ~ 6 months and >6 months between the two groups (72:8 vs. 64:4), the difference is not statistically significant (*p* > 0.05), suggesting that the efficacy time of two different brands of botulinum toxin A used in patients with Meige syndrome with anxiety symptoms is comparable, see Table 3.

**Table 3:** Ratio of duration of maintenance of efficacy in the two groups (*n*).

Index	Hengli group (*n* = 80)	Botox group (*n* = 68)	*χ*^2^ value	*p-*value
3 ~ 6 months	72	64	0.836	0.360
>6 months	8	4		

### 3.4 Comparison of SAS and SDS scores before and after treatment

SAS and SDS scores are used to assess the severity of anxiety and depression symptoms. As shown in Table 4, there was no significant difference in baseline SAS or SDS scores between the two groups (both *p* > 0.05). Three months after treatment, both groups showed significant reductions in SAS and SDS scores (all *p* < 0.001), indicating substantial improvement in anxiety and depression symptoms. However, there was no statistically significant difference between the two groups in post-treatment scores or in the magnitude of improvement (ΔSAS and ΔSDS, both *p* > 0.05).

**Table 4:** Comparison of SAS and SDS scores before and 3 months after treatment (x̅ ± s, points).

Group	*n*	SAS (pre)	SAS (post)	ΔSAS	SDS (pre)	SDS (post)	ΔSDS
Hengli group	80	55.32 ± 4.38	39.85 ± 4.32	−15.47 ± 2.10*	52.74 ± 4.16	38.22 ± 4.72	−14.52 ± 2.30*
Botox group	68	54.80 ± 4.45	40.27 ± 4.40	−14.53 ± 2.05*	52.58 ± 4.23	38.50 ± 4.88	−14.08 ± 2.15*
t-value (between groups, post)				0.715			0.231
*p*-value (between groups, post)				0.476			0.871

^*^p < 0.001 for within-group comparison (pre vs. post). Δ = post-treatment score minus pre-treatment score.

**Figure 1:**
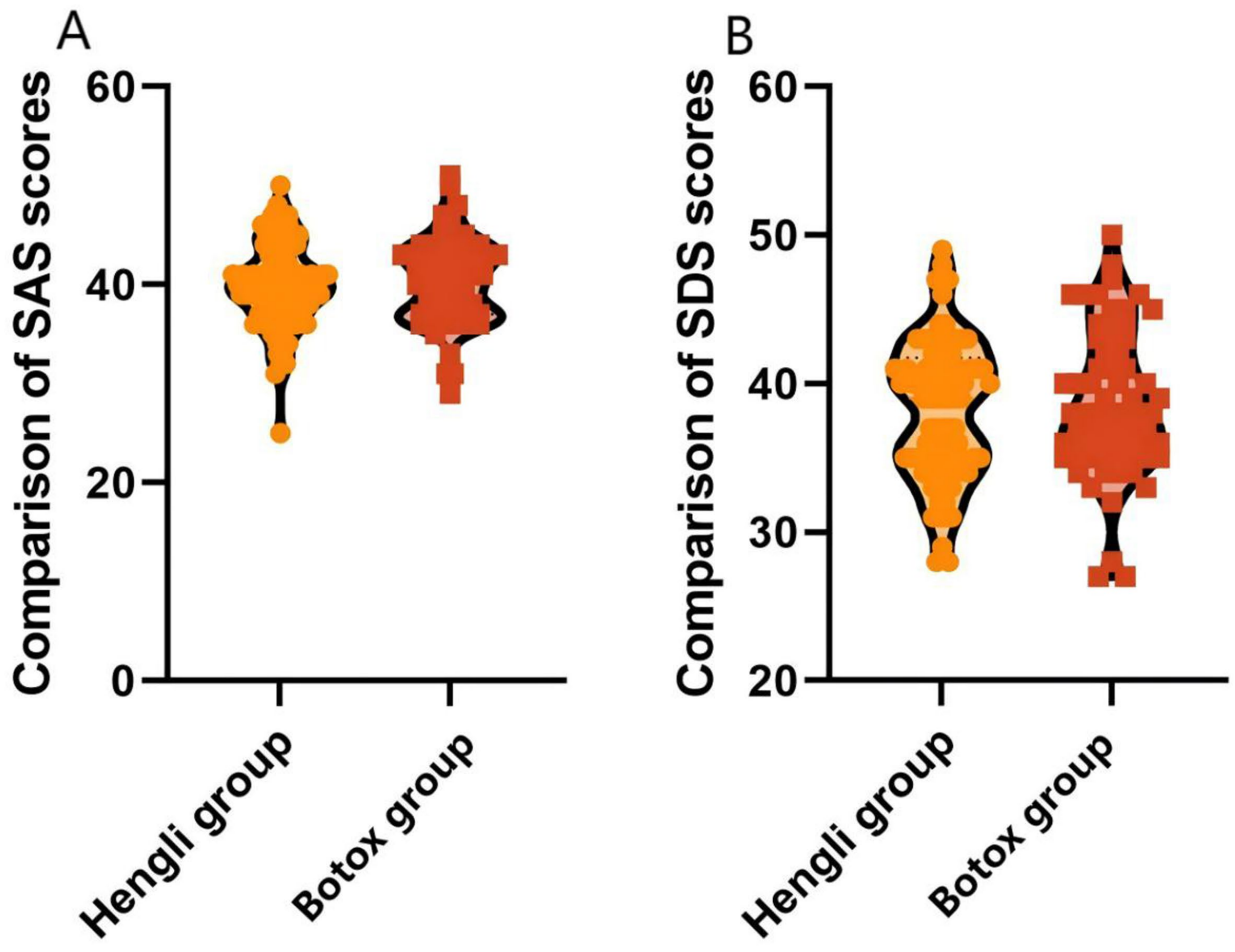
Comparison of SAS and SDS scores between the two groups of patients after treatment.

After treatment, the SAS (39.85 ± 4.32 vs. 40.27 ± 4.40) and SDS (38.22 ± 4.72 vs. 38.50 ± 4.88) scores of the Hennessey group and the BOTOX group were compared, and the difference was not statistically significant (*p*>0.05), indicating that the two different brands of botulinum toxin type A were effective in reducing anxiety and depression in patients with the accompanying Meige syndrome with anxiety symptoms. Figure 1 shows anxiety and depression in patients.

### 3.5 Comparison of polysomnographic parameters before and after treatment

Polysomnography parameters are used to monitor sleep quality. As shown in Table 5, there were no significant differences in baseline SE, TST, SL, AI, or REMS between the two groups (all *p* > 0.05). Three months after treatment, both groups showed significant improvements in all sleep parameters (all *p* < 0.001), including increased SE, TST, and REMS, and decreased SL and AI. However, there was no statistically significant difference between the two groups in any post-treatment parameter or in the magnitude of change (all *p* > 0.05).

**Table 5:** Comparison of polysomnographic parameters before and 3 months after treatment (x̅ ± s).

Parameter	Group	*n*	Pre-treatment	Post-treatment	ΔValue	t (between groups, post)	*p* (between groups, post)
SE (%)	Hengli	80	63.28 ± 10.24	68.45 ± 12.20	+5.17 ± 3.10*	0.082	0.934
	Botox	68	63.14 ± 10.36	69.10 ± 12.45	+5.96 ± 3.20*		
TST (min)	Hengli	80	302.55 ± 21.45	338.76 ± 24.65	+36.21 ± 8.50*	0.347	0.729
	Botox	68	303.78 ± 21.56	340.20 ± 25.18	+36.42 ± 8.70*		
SL (min)	Hengli	80	26.57 ± 4.20	22.35 ± 4.02	−4.22 ± 1.50*	0.299	0.766
	Botox	68	26.78 ± 4.34	21.80 ± 4.17	−4.98 ± 1.60*		
AI (times/h)	Hengli	80	37.50 ± 5.62	33.25 ± 5.65	−4.25 ± 1.80*	0.191	0.849
	Botox	68	37.68 ± 5.80	32.74 ± 5.78	−4.94 ± 1.90*		
REMS (min)	Hengli	80	7.40 ± 1.52	8.69 ± 1.54	+1.29 ± 0.60*	0.323	0.747
	Botox	68	7.32 ± 1.48	8.90 ± 1.60	+1.58 ± 0.65*		

^*^p < 0.001 for within-group comparison (pre vs. post). Δ = post-treatment value minus pre-treatment value.

**Figure 2:**
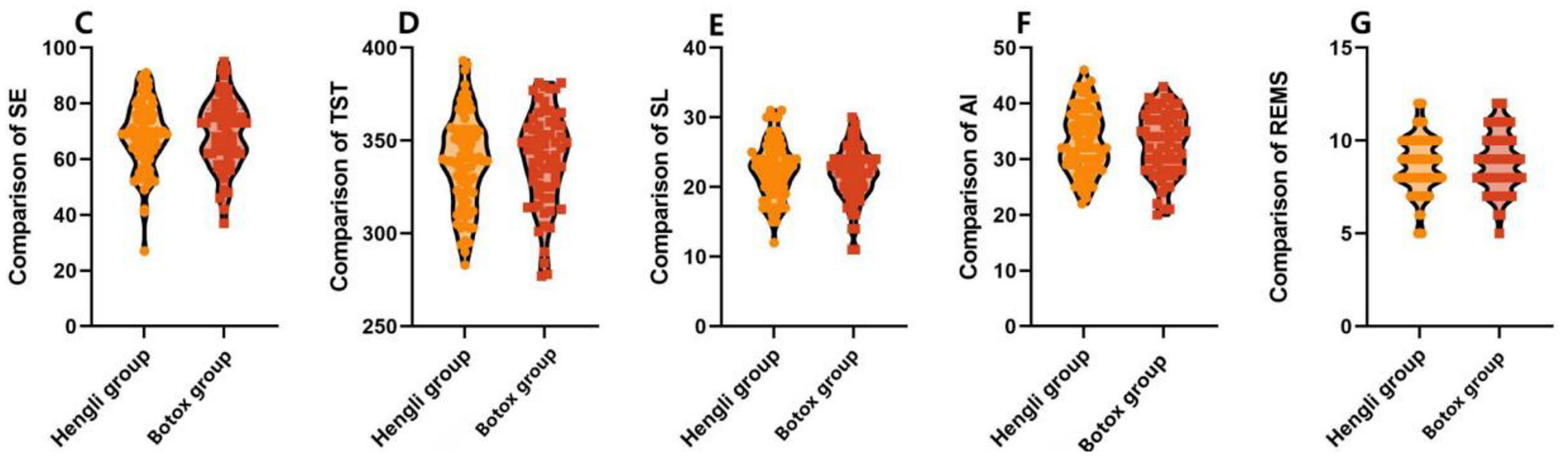
Comparison of polysomnographic monitoring parameters between the two groups of patients after treatment.

After treatment, SE (68.45 ± 12.20 vs. 69.10 ± 12.45) %, TST (338.76 ± 24.65 vs. 340.20 ± 25.18) min, SL (22.35 ± 4.02 vs. 21.80 ± 4.17) min, AI (33.25 ± 5.65 vs. 32. 74 ± 5.78) beats/min, and REMS (8.69 ± 1.54 vs. 8.90 ± 1.60) min were compared. The differences were not statistically significant (*p*>0.05), indicating that two different brands of botulinum toxin type A were effective in improving the quality of sleep in patients with Meige syndrome accompanied by symptoms of anxiety, as shown in Figure 2.

### 3.6 Comparison of the incidence of adverse reactions between the two groups of patients

The incidence of adverse reactions is used to assess the safety of the drug, and a low incidence of adverse reactions suggests high safety of the drug. The incidence of adverse reactions such as incomplete eyelid closure, facial muscle weakness, and drooping of the corners of the mouth during expression muscle activity was 5.00% in the Hengli group and 4.41% in the Botox group, with no statistically significant difference between the groups (*p* > 0.05). This indicates that the two different brands of botulinum toxin type A have a comparable safety profile in patients with Meige syndrome with anxiety symptoms, as shown in Table 6.

**Table 6:** Comparison of the incidence of adverse reactions between the two groups [*n* (%)].

Index	Hengli group (*n* = 80)	Botox group (*n* = 68)	*χ*^2^ value	*p-*value
Inadequate eyelid closure (*n*)	2	1		
Facial muscle Weakness (*n*)	1	1		
Drooping of the corners of the mouth during expression muscle activity (*n*)	1	1		
Total [*n* (%)]	4 (5.00)	3 (4.41)	0.028	0.867

### 3.7 Correlation analysis of injection parameters with clinical outcomes

Subgroup analysis based on injection patterns (e.g., inclusion of corrugator/procerus vs. orbicularis oculi/zygomaticus major) showed no significant difference in the improvement of SAS, SDS, or polysomnographic parameters between subgroups (all *p* > 0.05). Similarly, total dose (range 25–50 U per side) was not correlated with the degree of improvement in anxiety, depression, or sleep outcomes (Pearson’s correlation coefficients ranged from −0.12 to 0.10, all *p* > 0.05).

## 4 Discussion

Meige syndrome is mainly characterized by involuntary bilateral eyelid closure accompanied by involuntary and irregular contraction of the orofacial muscles, which can cause social disorders and inconvenience to the daily life of the patients, and some of the patients have anxiety and depression symptoms (20). Clinical studies have confirmed that patients with facial muscle spasm are often accompanied by anxiety and depression and other mood disorders, and the more severe the spasm, the more severe the mood disorders (21). At present, Meige syndrome with anxiety symptoms takes dopamine receptor antagonists; antidepressant drug treatment has a certain effect, but there are individual differences and adverse reactions (22). Therefore, the above treatments have certain limitations, and local injection of botulinum toxin type A is an effective treatment for Meige syndrome with anxiety symptoms, which has the advantages of quick effect, repeatability, and simple operation (23). In the present study, two different brands of local injection of botulinum toxin type A were found to be effective and safe for the treatment of Meige syndrome with anxiety symptoms and were comparable in terms of efficacy, duration of action, and improvement of psychological state and sleep quality, suggesting that both brands of botulinum toxin type A can be used in the treatment of Meige syndrome with anxiety symptoms.

The observed reductions in SAS and SDS scores (by approximately 15 points) represent a transition from moderate to mild anxiety/depression categories, which is clinically meaningful as it reflects not only statistical significance but also a perceptible improvement in patients’ emotional wellbeing. Similarly, improvements in polysomnographic parameters (e.g., increased sleep efficiency by ~5–6% and total sleep time by ~36 min) align with previously reported minimal clinically important differences in sleep outcomes for patients with movement disorders.

Regarding the differences between the two botulinum toxin type A products, Hengli (Chinese botulinum toxin type A) and onabotulinumtoxinA (Botox), it should be noted that while both are derived from *Clostridium botulinum* type A, they differ in molecular formulation, excipients, and unit potency definition. Hengli contains gelatin of porcine origin as a stabilizing agent, which may carry a theoretical risk of allergic reactions in sensitized individuals, whereas Botox uses human serum albumin. Both products are approved for therapeutic use in their respective regions (Hengli in China, Botox internationally for various neurological and aesthetic indications), but neither is specifically approved for Meige syndrome. The comparable efficacy and safety profiles observed in this study suggest that both formulations can be considered for off-label use in this condition, with choice depending on local availability, cost, and physician experience.

There are two brands of botulinum toxin type A currently used in Meige syndrome treatment in China: one is Hengli, produced in China, and the other is Botox, produced in the United States, both of which are purified from *Clostridium botulinum* type A. Currently, there are fewer clinical studies on the use of both brands of botulinum toxin type A for Meige syndrome. The results of this study showed that there was no significant difference in the overall relief rate between the two brands of botulinum toxin type A at 3 months after injection, indicating that both were effective in alleviating spasm symptoms in patients with Meige syndrome and anxiety. This is because both brands diffuse locally in the muscle after local injection and act rapidly on peripheral cholinergic nerve endings, triggering muscle relaxation and improving muscle spasticity (24). The antimuscarinic effect of botulinum toxin A on blocking acetylcholine on the nervous system is short-lived and reversible and usually lasts for only a few months (25). Although previous studies have shown that botulinum toxin type A remains effective even after more than 20 years of continuous injections, the efficacy of botulinum toxin type A may gradually diminish with the increase in the number of injections and the course of the disease, and the interval between injections is recommended to be more than 3 months (26). In this study, we compared the duration of efficacy of the two groups after injection and found that the difference in the duration of efficacy of the two brands of botulinum toxin A was not statistically significant, suggesting that the duration of efficacy of the two types of botulinum toxin A is comparable, and the efficacy of the two types of botulinum toxin A can be maintained for a period of 3–6 months.

It has been found in clinical work that patients with Meige syndrome usually suffer from anxiety as well as insomnia. Studies in the relevant literature have shown that 30 to 60% of patients with Meige syndrome have comorbid anxiety and depression, and the risk of anxiety and depression is higher than that of the normal population (27). Negative emotions such as anxiety and depression can attenuate adrenocortical function, and stress-related cortisol is closely related to dopamine levels in the striatum, which can stimulate the transient eye reflex and lead to blepharospasm, so it is inferred that the changes in dopamine grades due to anxiety and depression may be related to the condition of migratory facial myoclonus and Meige syndrome (28). The results of this study showed that the difference in SDS and SAS scores between the two groups after treatment was not statistically significant, suggesting that both types of A botulinum toxin can significantly improve patients’ anxiety and depression after local injection and that the improvement effect of the two is comparable. Both brands of A-type botulinum toxin can improve the psychological state of patients, mainly because both of the two drugs can reduce muscle spasm by blocking the release of acetylcholine at the neuromuscular junction after use to reduce the abnormal contraction of the facial muscles, which not only relieves the discomfort of the face but also reduces the emotional disorders caused by the pain and discomfort (29).

Recent studies on brain contact patterns have found that in addition to structural and functional changes in the basal ganglia, the pathobiochemical process of Meige syndrome involves structural and functional abnormalities in the basal ganglia, brainstem cerebellum, sensory-motor cortex, visual pathway, limbic system, etc., of which the dopaminergic pathway of the mesolimbic–limbic system is involved in sleep regulation, with the ventral pallidum and the nucleus ambiguus being the two important nuclei in this pathway (30). Disruption of the above anatomical structures and neurotransmitter transmission dysfunction can lead to sleep disorders. Polysomnographic parameters are commonly used in the assessment of sleep disorders, and the present study showed that there was no statistically significant difference in the grades of SE, TST, SL, AI, and REMS between the two groups before and after treatment, suggesting that both types of botulinum toxin A can significantly improve patients’ sleep disorders after topical injection, which may be because the two types of botulinum toxin can indirectly improve sleep quality by improving the function of the brain’s anatomical structure and regulating the grade of neurotransmitter expression through the mechanism of action. The reason may be that both of them can indirectly improve the quality of sleep through improving the function of brain anatomy and regulating the expression grade of neurotransmitters. In addition, this study found that no allergic or systemic reactions occurred in both groups, and the adverse reactions mainly included incomplete eyelid closure, facial muscle weakness, and ptosis, which were mild and could be relieved naturally, suggesting that both botulinum toxin type A are relatively safe.

Although the above research results were achieved in this paper, certain limitations still exist: For example, the small sample size included in this study and the fact that the samples all originated from a single hospital may lead to limited representativeness and generalization of the results; the follow-up time in the study was short, and the long-term efficacy of the patients concerned is still not completely clear; the retrospective study relied on the existing clinical databases and medical records, and there may be incomplete or missing data. This may affect the accuracy and reliability of the study results. Therefore, it is necessary to carry out large-sample, multicenter studies in the future, extend the follow-up period to obtain long-term benefits, strengthen the quality control management of data organization, and collect as much clinical data as possible to provide more reliable and substantive support for clinical treatment planning.

## 5 Conclusion

In this retrospective cohort study, local injection of botulinum toxin A is safe and effective for Meige syndrome with anxiety symptoms, and there is no significant difference in the efficacy, duration of action, and improvement of psychological state and sleep quality between different brands of botulinum toxin A. Clinical practice should be based on the actual situation and then reasonably select the appropriate brand of botulinum toxin A to carry out treatment.

## Data Availability

The raw data supporting the conclusions of this article will be made available by the authors, without undue reservation.
